# Bullous pellagra in a patient with alcohol use disorder and gastric bypass

**DOI:** 10.1016/j.jdcr.2026.05.050

**Published:** 2026-05-27

**Authors:** George Ladas, Joseph Cascone, Ahmed H. Badawi

**Affiliations:** a Kansas City University, Joplin, Missouri; b School of Medicine, University of Missouri-Kansas City, Kansas City, Missouri; c Division of Dermatology, The University of Kansas Medical Center, Kansas City, Kansas; d Freeman Health System Dermatology and Skin Cancer Center, Joplin, Missouri

**Keywords:** bullous dermatosis, niacin deficiency, nutritional deficiency, pellagra

## Introduction

Pellagra is a chronic wasting disorder due to niacin (vitamin B3) deficiency.^1^ It classically presents with a triad of dermatitis, dementia, and diarrhea. Skin manifestations of pellagra include photodistributed hyperpigmentation, erosions, and classically, a circular collar rash known as Casal’s necklace.^2^ Patients can present with glossitis, diarrhea, vomiting, dementia, and hallucinations.^3^ Pellagra results from niacin deficiency or impaired tryptophan metabolism, which may arise from inadequate dietary intake, malabsorption, chronic alcoholism, or genetic conditions such as Hartnup disease.^4^ Nowadays, pellagra is most commonly encountered in individuals with chronic alcohol use disorder, malnutrition, or in regions where untreated maize (corn) constitutes a dietary staple.^5^ If not corrected, niacin deficiency can lead to multiorgan failure and death.^6^ Here, we described a case of a female patient with pellagra initially misdiagnosed as erythema multiforme.

## Case description

A 31-year-old woman with a history of alcoholic pancreatitis, alcohol use disorder, bariatric surgery, and mood disorder presented to the hospital with a chief complaint of an erosive rash on her extremities. The rash started on her right dorsal upper extremity 10 days prior and spread proximally up her arm and bilateral lower extremities. She reported the rash was painful with a burning sensation. She endorsed nausea, vomiting, and increased sun exposure prior to rash onset but denied abdominal pain, vision changes, genital sores, sore throat, fever, or joint pain. She denied use of new medications, supplements, or exposure to caustic chemicals prior to rash onset. Her only medication was 200 mg of quetiapine twice daily. Prior to the current hospitalization, she was initially evaluated at a different hospital 1 week earlier, where she was treated for presumed erythema multiforme with valacyclovir and an oral prednisone taper, as her initial skin biopsy demonstrated full-thickness epidermal necrosis with fragments of detached epidermis. She was discharged because, at the time, the rash was unprogressive.

**Fig 1:**
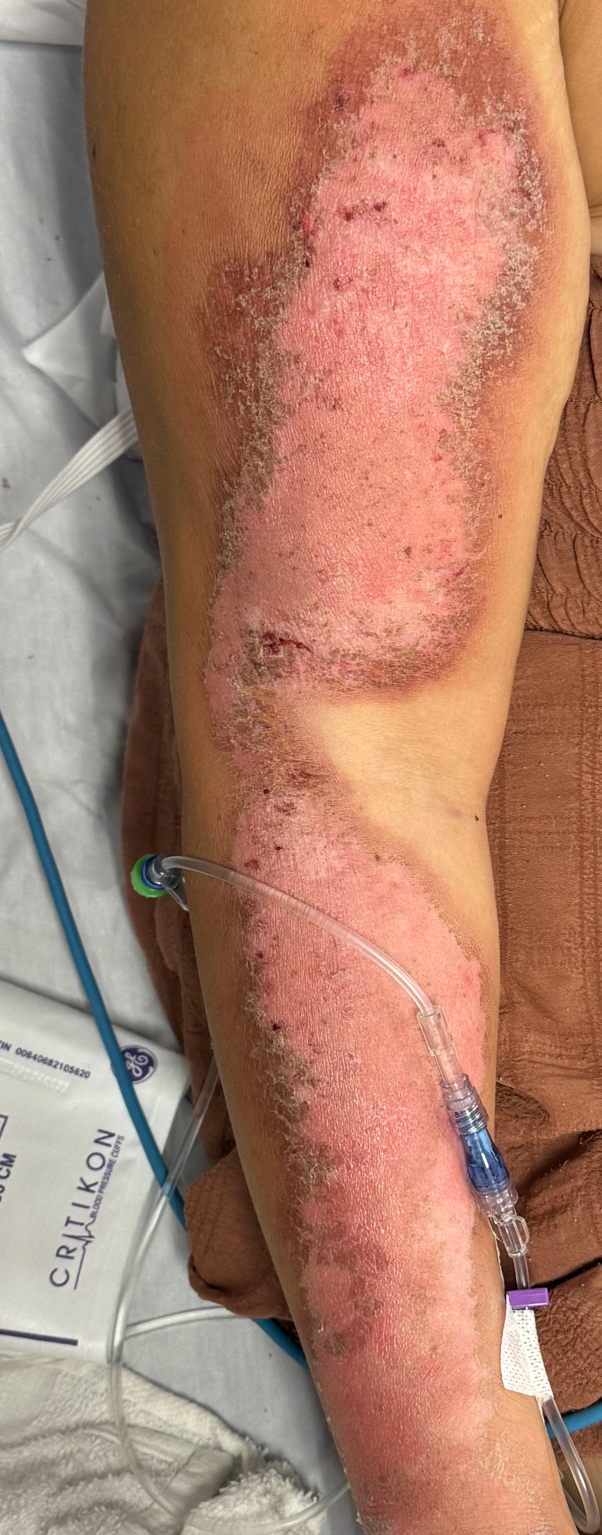
Clinical image demonstrating the photodistributed eroded rash on the right upper extremity.

**Fig 2:**
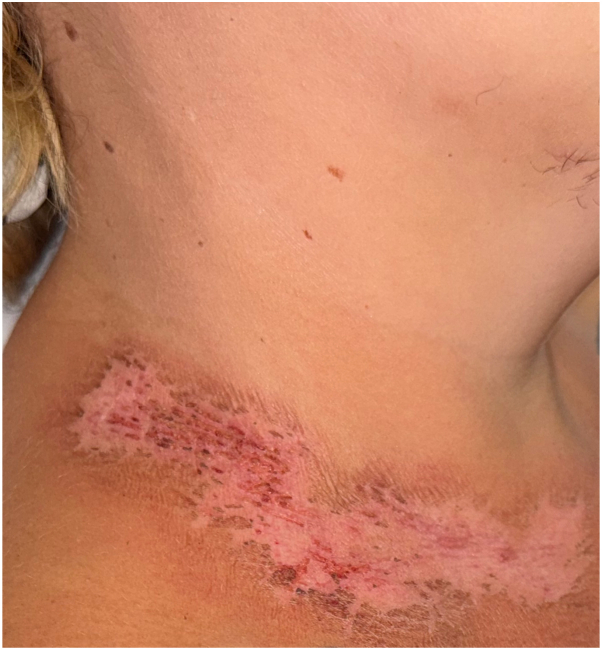
Clinical image demonstrating classic Casal’s sign of pellagra.

When her rash did not improve, she presented to our hospital. The physical examination revealed a nondistended abdomen, no palpable cervical or supraclavicular lymphadenopathy, and an intact neurologic examination. Dermatologic examination showed confluent erythematous and faintly violaceous eroded patches with peripheral scale on the right side of the neck, anterior portion of the right upper extremity with sparing of the medial antecubital fossa (Fig 1), and right lower extremity. The rash was sharply demarcated on the dorsal aspect of the right foot and right lower extremity. Violaceous patches on the left knee, dorsal aspect of the left foot, and upper portion of the left arm with some central vesiculation and superficial central erosion were also present. Nikolsky's sign was negative, and Casal’s collar sign was positive (Fig 2) with normal conjunctivae and oral mucosa. The differential diagnosis included pellagra, phototoxic drug eruption, severe phytophotodermatitis, bullous lupus, porphyria, pseudoporphyria, and generalized bullous fixed drug eruption.

Punch biopsies of the left extremity revealed keratinocyte vacuolation and pallor with hyperpigmentation, evolving full-thickness epidermal necrosis, subepidermal bulla formation, and re-epithelialization. No definitive interface reaction or dermal inflammatory infiltrate was present. Laboratory test results revealed hypoalbuminemia at an albumin level of 2.5 g/dL, macrocytic anemia with a red blood cell count of 2.41 million cells/μL and mean corpuscular volume of 111.6 μm^3^, mild hyponatremia at an Na level of 135 mEq/L, and hyperglycemia at a blood glucose level of 126 mg/dL. Serum levels of nicotinamide and zinc were obtained and found to be low at < 20 ng/mL and 56 μg/dL, respectively. These findings in conjunction with the history and examination suggested pellagra as well as zinc deficiency.

The patient was given wound care to eroded areas, including daily bacitracin. She was started on intravenous fluids; thiamine; and nutritional support, including a bariatric multivitamin, niacinamide 500 mg, and zinc sulfate 220 mg daily. She was counseled on strict photoprotection and cessation of alcohol use. Her symptoms of burning and fatigue improved, and her neck rash was almost completely resolved by discharge time.

## Discussion

The patient in the case above presented with bullae and rapid skin degeneration, initially suggestive of erythema multiforme. The cutaneous lesions in pellagra typically consist of early erythema, occasionally with vesicles or bullae, followed by hyperkeratosis and scaly hyperpigmentation.^7^ The pronounced vesicles and bullae, along with an initial biopsy with findings suggestive of interface dermatitis, contributed to a previous misdiagnosis of erythema multiforme. The antibiotics and steroids slowed the inflammatory components of the cutaneous eruption; however, the rash spread with typical niacin deficiency progression, worsening due to subsequent sun exposure. UV exposure worsens pellagra due to nicotinamide adenine dinucleotide impairing DNA repair and skin cell recovery.^5^ Characteristic histologic findings of pellagra include dilated dermal blood vessels with minimal inflammation, epidermal pallor and keratinocyte ballooning, compact or parakeratotic hyperkeratosis, and subepidermal bullae.^5^ Early histologic changes in niacin deficiency can be subtle and nonspecific.^5^ With progression, these changes evolve into more characteristic pellagra findings.

Bariatric surgery increases patients' risk of nutritional deficiencies.^8^ Rare in the United States, niacin deficiency should be considered in malnourished patients with alcohol use or bariatric surgery presenting with fatigue, nausea, and dermatitis.^9^ Cutaneous manifestations of nutritional deficiencies are important recognition tools for both patients and clinicians. Adherence to supplementation guidelines after surgery is essential for long-term health. Of note, it is important that when a nutritional deficiency is identified, a comprehensive work-up is completed to identify any other deficiencies. As in this case, patients can commonly have multiple nutritional deficiencies concurrently.

For patients presenting with nonspecific, progressive photodistributed dermatitis accompanied by gastrointestinal and neurologic symptoms with a history of bariatric surgery and alcohol abuse, pellagra should be considered in the differential diagnosis. Existing literature underscores the importance of timely diagnostic evaluation to mitigate the fatal complications associated with untreated niacin deficiency.

## Conflicts of interest

None disclosed.
